# Re-operation rate after surgical treatment of osteochondral lesions of the talus in paediatric and adolescent patients

**DOI:** 10.1186/s13018-021-02282-z

**Published:** 2021-03-15

**Authors:** Daniel Körner, Christoph E. Gonser, Stefan Döbele, Christian Konrads, Fabian Springer, Gabriel Keller

**Affiliations:** 1 Department of Traumatology and Reconstructive Surgery, BG Trauma Center Tübingen, Eberhard Karls University Tübingen, Schnarrenbergstr. 95, 72076 Tübingen, Germany; 2 Department of Diagnostic and Interventional Radiology, University Hospital Tübingen, Eberhard Karls University Tübingen, Hoppe-Seyler-Str. 3, 72076 Tübingen, Germany; 3 Department of Radiology, BG Trauma Center Tübingen, Eberhard Karls University Tübingen, Schnarrenbergstr. 95, 72076 Tübingen, Germany

**Keywords:** Osteochondral lesion, Osteochondritis dissecans, Ankle, Talus, Children, Paediatric, Pediatric, Bone marrow stimulation, Re-operation, Retrograde drilling

## Abstract

**Background:**

The aim of this study is to analyse the re-operation rate after surgical treatment of osteochondral lesions of the talus (OCLTs) in children and adolescents.

**Methods:**

Between 2009 and 2019, 27 consecutive patients with a solitary OCLT (10 male, 17 female; mean age 16.9 ± 2.2 years; 8 idiopathic vs. 19 traumatic) received primary operative treatment (arthroscopy + bone marrow stimulation [BMS], *n* = 8; arthroscopy + retrograde drilling, *n* = 8; autologous chondrocyte implantation [ACI]/autologous bone grafting, *n* = 9; arthroscopy + BMS + retrograde drilling; *n* = 1; flake fixation, *n* = 1). Seventeen OCLTs were located at the medial and ten at the lateral talus.

‘Re-operation’ as the outcome measure was evaluated after a median follow-up of 42 months (range 6–117 months). Patients were further subdivided into groups A (re-operation, *n* = 7) and B (no re-operation, *n* = 20). Groups A and B were compared with respect to epidemiological, lesion- and therapy-related variables.

**Results:**

Seven of 27 patients needed a re-operation (re-operation rate 25.9% after a median interval of 31 months [range 13–61 months]). The following operative techniques were initially used in these seven patients: arthroscopy + BMS *n* = 2, arthroscopy + retrograde drilling *n* = 4, ACI + autologous bone grafting *n* = 1. A comparison of group A with group B revealed different OCLT characteristics between both groups. The intraoperative findings according to the International Cartilage Repair Society (ICRS) classification revealed significantly more advanced cartilage damage in group B than in group A (*p* = 0.001).

**Conclusions:**

We detected a re-operation rate of 25.9% after primary surgical OCLT treatment. Patients with re-operation had significantly lower ICRS classification stages compared to patients without re-operation.

## Introduction

Osteochondral lesion of the ankle (OCLA) is a descriptive term for lesions affecting the articular cartilage and/or the subchondral bone of the talus or tibial plafond. This term encompasses different lesion types according to aetiology and pathophysiology. OCLA may be associated with ankle trauma; the reported rates of cartilage defects following acute ankle sprains are approximately 50% [1, 2]. Furthermore, there is an association between chronic ankle instability and OCLA [3, 4]. Moreover, OCLA also occur without a trauma to the affected ankle in the patient’s history (idiopathic) [5]. However, the conceptual distinction between the terms OCLA and *osteochondritis dissecans* is not clear; *osteochondritis dissecans* more likely represents a subgroup of OCLA.

OCLA are very uncommon in preschool children but more frequent in school children, especially in the female adolescent population [6]. The reported incidence of OCLA in Southern California is 4.6 per 100,000 in 6–19-year-olds, and the female-to-male ratio is 1.6:1 [6].

There are no treatment guidelines for OCLA in children, and the therapeutic management of OCLA in adults is also used in children. Good results with conservative treatment can be achieved in stable lesions (up to stage III, according to the Berndt-Harty-Loomer [BHL] classification) [5]. The intra-articular injection of platelet-rich plasma or hyaluronic acid represent promising new therapeutic approaches in the treatment of OCLA [7]. Failed conservative treatment, loose bodies, unstable lesions, subchondral bone sclerosis and BHL stage advancement are considered to be indications for surgical treatment [5].

There are various arthroscopic and open surgical methods available for treating OCLA [8, 9]. The decision for a surgical procedure depends on whether only cartilage or subchondral bone or both are affected; furthermore, the size and location of the OCLA are relevant. Operative techniques include fixation of (osteo-) chondral flakes in acute cases, debridement of the lesion, bone marrow stimulation (BMS; e.g. micro- and nano-fracturing and drilling) or retrograde drilling/bone grafting of a bony defect in case of intact overlaying cartilage. Regenerative strategies for larger lesions (> 1.5 cm^2^) are autologous chondrocyte implantation (ACI) and matrix-induced bone marrow stimulation (M-BMS). Both techniques can be combined with bone grafting for subchondral defects. Osteochondral transplantation is also reserved for large lesions [9–12]. Concomitant malalignment of the ankle and ankle instability should be evaluated and integrated into the treatment plan [10, 13].

Reilingh et al. analysed the clinical and radiographic outcome of 36 children (mean age = 13 years) with an osteochondral lesion of the talus (OCLT) after conservative and operative treatment [14]. The authors reported a high failure rate of conservative treatment, with four of seven patients eventually requiring surgery after initial conservative treatment.

Kramer et al. analysed the results of surgical treatment of 109 ankles in 100 paediatric OCLA patients with a mean age of 14.3 years after a median follow-up of 3.3 years [15]. The authors found a re-operation rate of 27%. Furthermore, female gender and higher body mass index (BMI) were associated with worse functional outcomes.

There is limited research on the influencing factors of the success of operative treatment of OCLA in the paediatric and adolescent populations. It is likely that epidemiological factors, lesion-related factors and therapy have an influence on the clinical outcome.

The aim of this study was to analyse the re-operation rate after initial surgical treatment of OCLTs in children and adolescents. Against the background of a translational approach in orthopaedics [16], this work represents a feedback from clinical application to basic research (from bedside to bench).

## Methods

### Study population

Between 2009 and 2019, 31 consecutive paediatric and adolescent patients received primary operative treatment of an OCLT at a single centre. The patient files and the imaging data of these patients digitally available in the picture archiving and communication system were retrospectively analysed.

The following inclusion criteria were defined: presence of a solitary OCLT, primary operative treatment at our institution and patient age 20 years or less. The following exclusion criteria were defined: available follow-up under 6 months. In total, 27 patients fulfilled the criteria. The median follow-up was 42 months (range 6–117 months).

The patients were treated with arthroscopy + BMS (*n* = 8), arthroscopy + retrograde drilling (*n* = 8), arthroscopy + BMS + retrograde drilling (*n* = 1), arthrotomy + flake fixation (*n* = 1), ACI + autologous bone grafting (*n* = 9). In two cases of BMS, an additional stabilisation of the ankle was performed. The therapy decision was based on several factors, such as the aetiology of the OCLT, course of treatment, clinical examination findings, lesion size/depth and cartilage damage (preoperative imaging and intraoperative findings), surgeon experience and preference, patient preference and patient compliance.

Table 1 gives a general overview of the most commonly used surgical techniques in our study with advantages and limitations.

**Table 1:** Comparison of three surgical techniques in the treatment of OCLA

	Arthroscopy, BMS	Retrograde drilling	ACI
Advantages	- Indicated for chondral and osteochondral lesions [17] - Reported reduction of pain and improvement of function (e.g. AOFAS score) [9] - Low complication rate [9] - Re-BMS is possible [17]	- Indicated for subchondral lesions [9] - Can be performed with intraoperative imaging and/or targeting device [18] - Good reported outcomes in patients with open growth plates and when performed as first-line surgical intervention [9] - Can be combined with bone grafting (lesion diameter > 1 cm, depth > 1 cm, cysts > 100 mm^3^) [18]	- Indicated for lesions with size > 1 cm^2^ with or without cysts [19]; vs other recommendations: lesion size ≥ 1.5 cm^2^ [20] - Good reported functional outcomes, even when performed after failed first-line surgical treatment [9] - Can be combined with bone grafting
Limitations	- Formation of fibrocartilage instead of hyaline cartilage [9] - Lesions < 10 mm in diameter, < 100 mm^2^ in area and < 5 mm in depth [17]; vs. other recommendations: lesion size < 1.5 cm^2^ [20]	- Overlaying cartilage must be intact to achieve good outcome [9, 18]	- Two-step procedure (harvesting and implantation) [9] - Harvest-site morbidity - Open procedure might be needed for implantation

*OCLA* osteochondral lesion of the ankle, *BMS* bone marrow stimulation, *ACI* autologous chondrocyte implantation, *AOFAS* American Orthopaedic Foot and Ankle Society

### Data capture

Epidemiological data and lesion-related variables were captured. The aetiology of the OCLT was classified as *acute traumatic* when there was a history of trauma within 6 weeks prior to diagnosis on imaging and as *chronic traumatic* when there was a history of trauma to the affected ankle longer than 6 weeks prior to the diagnosis. The OCLT was classified as *idiopathic* when there was no history of trauma to the ankle in the medical history of the patient.

The OCLTs were classified according to the International Cartilage Repair Society (ICRS) classification, BHL classification and Magnetic Resonance Observation of Cartilage Repair Tissue score (MOCART 2.0) [21]. In this study, we report the ICRS classification according to preoperative and postoperative magnetic resonance imaging (MRI) and the intraoperative findings separately. The stage was evaluated according to the BHL classification [22] on the basis of preoperative imaging (including conventional radiographs, computed tomography [CT] and MRI).

Preoperative as well as postoperative MRI scans were evaluated by a single experienced musculoskeletal radiologist (senior author) blinded to patient history. Lesion size (area) and depth were determined using the preoperative MRI findings; the size was calculated by multiplying the length by the width. The MOCART 2.0 score was calculated based on the postoperative MRI.

The type of operation and the operation date for all primary operations and re-operations were captured. In case of ACI, the prior operation for chondrocyte harvesting and implant removal after ACI were not counted as separate operations when there was no cartilage therapy applied.

### Outcome measures

Re-operation after initial operative treatment at our institution was defined as the outcome measure. Re-operation was performed if the patients still had symptoms or had symptoms again and pathologies were found on re-imaging. For this analysis, the patients were contacted and asked whether they had undergone re-operation after treatment at our institution. For patients who could not be contacted, we defined the date of the last treatment at our institution as the last follow-up.

### Statistical analysis

Statistical analysis was performed using the software package JMP (SAS Institute Inc., JMP, Version 12.2.0, Cary, NC, USA). The Shapiro-Wilk W test was applied to screen the data for normality of distribution. Mean and standard deviation for normally distributed data and median and range for non-normally distributed data were reported.

The study population was further subdivided into groups A and B; patients in group A (*n* = 7) had undergone re-operation, and patients in group B (*n* = 20) had not undergone re-operation to the best of our knowledge. The two groups were compared according to demographics and lesion- and therapy-related variables as well as MRI findings. The Chi-square test was used for categorical data. For continuous data, the Wilcoxon test was used for non-normally distributed data, and the *t* test was used for normally distributed data. To compare the two groups according to the primary operative techniques used, the operative techniques were divided into four groups: ‘BMS’ (*n* = 8), ‘retrograde drilling’ (*n* = 8), ‘ACI’ (*n* = 9) and ‘others’ (*n* = 2).

The level of significance was set at *p* = 0.05 for all statistical tests.

## Results

Within the study population, the majority of OCLTs were located at the medial talus (17/63.0% medial vs. 10/37.0% lateral). All lateral OCLTs were associated with a trauma to the affected ankle (3 acute and 7 chronic traumatic) in contrast to medial OCLTs (idiopathic: 8, acute traumatic: 4, chronic traumatic: 5). Medial OCLTs had a mean size on preoperative MRI of 1.27 ± 0.52 cm^2^ in contrast to lateral OCLTs with a mean size of 1.04 ± 0.31 cm^2^. The mean lesion size on preoperative MRI according to aetiology was 1.17 ± 0.31 cm^2^ in idiopathic, 1.20 ± 0.39 cm^2^ in acute traumatic and 1.23 ± 0.61 cm^2^ in chronic traumatic OCLTs.

Seven of 27 patients needed a re-operation (re-operation rate 25.9%). The following operative techniques were initially used in these seven patients: two patients received arthroscopy with BMS, four arthroscopy with retrograde drilling and one ACI with autologous bone grafting. The median time between the primary operation and the re-operation was 31 months (range 13–61 months).

Regarding the operative technique in the re-operation, four patients received ACI, two of which were with autologous bone grafting. The patient who initially had ACI with autologous bone grafting had undergone arthroscopy with scare debridement and cell harvesting for re-ACI as re-operation; however, the re-ACI was not performed in the further course. One patient had undergone diagnostic arthroscopy with Broström procedure as re-operation, and the other patient had undergone arthroscopy with osteophyte debridement and re-BMS of the same OCLT.

The comparison between group A (re-operation; *n* = 7) and group B (no re-operation; *n* = 20) is shown in Table 2. The age and gender distribution and BMI of patients within the two groups were not significantly different. The majority of OCLTs within both groups were located medially and were of traumatic aetiology (acute or chronic traumatic; group A: 71.5% vs. group B: 70.0%). The majority of cases within both groups were BHL stage 2 or 3. The intraoperative findings according to the ICRS classification revealed significantly more advanced cartilage damage within group B than within group A (group A: 16.7% ICRS stage 4 vs group B: 68.4% ICRS stage 4; *p* = 0.001).

**Table 2:** Comparisons between group A (re-operation) and group B (no re-operation)–demographics

	Group A *n* = 7	Group B *n* = 20	*P* value/test
Age (Mean ± SD)	16.3 ± 1.6 years	17.1 ± 2.4 years	n.s.^c^
Gender (*n*/%)			
Male	2/28.6	8/40.0	n.s.^b^
Female	5/71.4	12/60.0	
BMI (Median, range)	21.7 kg/m^2^ (20.3–34.9 kg/m^2^)	23.6 kg/m^2^ (18.0–39.3 kg/m^2^)	n.s.^a^
Lesion location			
Medial	5/71.4	12/60.0	n.s.^b^
Central	0	0	
Lateral	2/28.6	8/40.0	
Corresponding distal tibia lesion (*n*/%)			
Yes	0	1/5.0	n.s.^b^
No	7/100.0	19/95.0	
Growth plate distal tibia/fibula (*n*/%)			
Open	0	1/5.0	
Closed	7/100.0	19/95.0	
Side (*n/*%)			
Right	4/57.1	9/45.0	n.s.^b^
Left	3/42.9	11/55.0	
Aetiology (*n*/%)			
Idiopathic	2/28.6	6/30.0	n.s.^b^
Acute traumatic	2/28.6	5/25.0	
Chronic traumatic	3/42.9	9/45.0	
BHL classification (*n*/%)			
1	0	2/10.0	n.s.^b^
2	2/28.6	5/25.0	
3	4/57.1	10/50.0	
4	1/14.3	3/15.0	
5	0	0	
ICRS classification intraoperative (*n*/%) Missing data *n* = 2			
0	0	0	0.001^b^
1	1/16.7	5/26.3	
2	2/33.3	1/5.3	
3	2/33.3	0	
4	1/16.7	13/68.4	
Operative technique (*n*/%)			
Arthroscopy, BMS	2/25.0	6/75.0	–
Arthroscopy, retrograde drilling	4/50.0	4/50.0	
Arthroscopy, BMS + retrograde drilling	0	1/100.0	
Arthrotomy, flake fixation	0	1/100.0	
ACI + autologous bone grafting	1/11.1	8/88.9	

*BMI* body mass index, *ICRS* International Cartilage Repair Society, *BHL* Berndt-Harty-Loomer, *BMS* bone marrow stimulation, *ACI* autologous bone grafting, *n*.*s*. not significant; ^a^ Wilcoxon test; ^b^ Chi square test; ^c^ *t* test

Table 3 shows the preoperative MRI findings. The size and depth of the OCLTs on preoperative MRI were not significantly different between group A and B. Group B contained a wider spectrum of stages according to the ICRS classification based on preoperative MRI and also more advanced OCLTs.

**Table 3:** Comparisons between group A (re-operation) and group B (no re-operation)–preoperative MRI

	Group A *n* = 7	Group B *n* = 20	*P* value/test
Lesion size (Mean ± SD) Not applicable *n* = 2	1.06 ± 0.36 cm^2^	1.25 ± 0.51 cm^2^	n.s.^c^
Lesion depth (Mean ± SD) Not applicable *n* = 2	4.4 ± 1.1 mm	5.1 ± 1.7 mm	n.s.^c^
ICRS classification (*n*/%)			
0	0	2/12.5	n.s.^b^
1	1/20.0	4/25.0	
2	3/60.0	2/12.5	
3	1/20.0	4/25.0	
4	0	4/25.0	
Joint effusion (*n*/%)			
Yes	4/80	13/81.3	n.s.^b^
No	1/20	3/18.7	
Bone marrow oedema (*n*/%)			
Yes	5/100.0	15/93.7	n.s.^b^
No	0	1/6.3	
Change of bony contour (*n*/%)			
Yes	2/40.0	11/68.7	n.s.^b^
No	3/60.0	5/31.3	
Associated subchondral cyst (*n*/%)			
Yes	1/20.0	3/18.8	n.s.^b^
No	4/80.0	13/81.2	
Fluid surrounding loose body (*n*/%)			
No	1/20.0	4/25.0	n.s.^b^
Incomplete	4/80.0	11/68.7	
Complete	0	1/6.3	
Dislocation of loose body (n/%)			
Yes	0	1/6.3	n.s.^b^
No	5/100.0	15/93.7	

Preoperative MRI was available in 21 cases at the time of retrospective analysis. *MRI* magnetic resonance imaging, *ICRS* International Cartilage Repair Society, *MOCART* magnetic resonance observation of cartilage repair tissue, *SD* standard deviation, ^b^ Chi square test; ^c^ t test

The postoperative MRI findings according to the ICRS classification as well as the MOCART 2.0 score were not significantly different (see Table 4); however, patients in group A showed a slightly higher MOCART 2.0 score.

**Table 4:** Comparisons between group A (re-operation) and group B (no re-operation)—postoperative MRI

	Group A *n* = 7	Group B *n* = 20	P value/test
ICRS classification (*n*/%)			
0	1/16.7	2/18.2	n.s.^b^
1	1/16.7	4/36.4	
2	3/50.0	2/18.2	
3	1/16.7	3/27.3	
4	0	0	
MOCART 2.0 score (Mean ± SD)	54.2 ± 21.3	45.0 ± 17.7	n.s.^c^
Joint effusion (*n*/%)			
Yes	4/66.7	7/63.6	n.s.^b^
No	2/33.3	4/36.4	
Bone marrow oedema (*n*/%)			
Yes	6/100.0	11/100.0	n.s.^b^
No	0	0	
Change of bony contour (*n*/%)			
Yes	3/50.0	8/72.7	n.s.^b^
No	3/50.0	3/27.3	
Associated subchondral cyst (*n*/%)			
Yes	2/33.3	4/36.4	n.s.^b^
No	4/66.7	7/63.6	

Postoperative MRI was available in 17 cases at the time of retrospective analysis. The median time between operation and last MRI was 13 months (range 2–73 months). *MRI* magnetic resonance imaging, *ICRS* International Cartilage Repair Society, *MOCART* Magnetic Resonance Observation of Cartilage Repair Tissue, *SD* standard deviation, ^b^ Chi square test; ^c^ t test

**Fig. 1:**
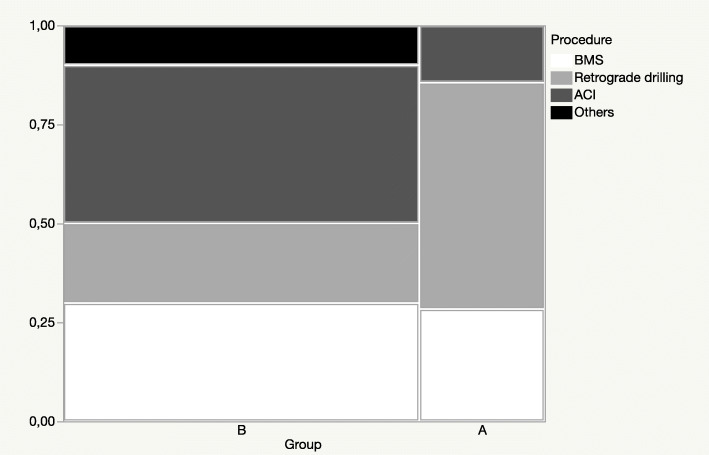
Mosaic plot showing the frequencies of the four procedure groups ‘BMS’ (*n* = 8), ‘retrograde drilling’ (*n* = 8), ‘ACI’ (*n* = 9) and ‘others’ (*n* = 2) within group A (re-operation; *n* = 7) and group B (no re-operation; *n* = 20), Chi-square test: *p* = 0.246; *ACI* autologous chondrocyte implantation, *BMS* bone marrow stimulation

When all primary procedures were grouped into the four groups ‘BMS’, ‘retrograde drilling’, ‘ACI’ and ‘others’, the distribution of the procedures was not significantly different between groups A and B (*p* = 0.246; Fig. 1). Within the group ‘BMS’, two of eight (25.0%) patients received a re-operation, within the group ‘retrograde drilling’, four of eight (50.0%) patients, and, within the ‘ACI’ group one of nine (11.1%).

## Discussion

The major findings of our study were that patients who needed a re-operation had lower-stage OCLTs—according to the intraoperative ICRS classification—than patients who did not need a re-operation. The re-operation rate was 25.9%; the patients who needed a re-operation were mainly treated with less invasive techniques, such as retrograde drilling or BMS. The surgical technique used initially certainly has a major influence on the re-operation rate because patients who were initially operated with a less invasive technique can be offered a more invasive technique if a re-operation is necessary. Patients who were initially operated with a more invasive technique have fewer alternatives in the event of a re-operation.

Kramer et al. also analysed the re-operation rate in a retrospective series of 109 OCLAs (107 OCLTs and 2 osteochondral lesions of the distal tibia) of 100 patients with a mean patient age of 14.3 ± 2.3 years and a median follow-up period of 3.3 years [15]. The most common procedures were transarticular drilling (54%), fragment fixation (20%) and excision microfracture (26%). They found a re-operation rate of 27%, which is comparable to our results. The reported median time between the initial operation and the re-operation was 1.7 years, whereas the median time was 31 months in our study. The authors found that the re-operation rate was significantly higher for OCLA with poor results on conventional radiographs [15].

A recent case series of eight skeletally immature patients (mean age 11.1 years) who were treated with retrograde drilling reported a significant improvement of clinical outcome (according to the Japanese Society for Surgery of the Foot scale) [23]; however, on the basis of follow-up CT, the healing was reported to be ‘good’ in four cases, ‘fair’ in three cases and ‘poor’ in one case. They performed retrograde drilling only in the case of intact overlaying cartilage identified in arthroscopy. Another series of eight patients (mean age 14.9 years) who were treated with retrograde drilling were also reported to have significant clinical improvements (according to the American Orthopaedic Foot and Ankle Society ankle hind-foot score and ankle activity score) [24]. The OCLTs treated in the above study had a cartilage status of ICRS stage 0 or 1. In contrast, because the patients in our population were older, the physis of the distal tibia and fibula were closed in the majority of patients. Our findings indicate that retrograde drilling in low-stage OCLTs is associated with a high re-operation rate in paediatric and adolescent patients. However, our findings do not explain how a re-operation could be avoided in these patients (i.e. conservative treatment or more aggressive surgical treatment) because doing so was not the objective of the present study. When interpreting the results, it must be considered that in patients with high-stage OCLTs who primarily receive ACI plus bone grafting, if symptoms persist at the same OCLT, less invasive, cartilage-preserving surgical techniques are no longer feasible. This could have influenced the re-operation rate in this group in our study.

Regarding demographics and lesion characteristics, our findings are consistent with those reported in the literature: The majority of patients were female (63.0%), and the majority of OCLTs were located at the medial talus (63.0%) [1, 6, 14]. Remarkably, the rate of laterally located OCLTs was higher in patients with a trauma to the affected ankle; all patients with idiopathic OCLTs in our study had medial OCLTs, whereas patients with acute traumatic and chronic traumatic OCLTs showed both lateral and medial OCLTs (lateral vs. medial: 42.9% vs. 57.1% and 58.3% vs. 41.7%, respectively).

There are various classification systems used for OCLTs [25]. Berndt and Harty established a classification system for transchondral fractures of the talus in 1959 [26], which was revised by Loomer in 1993 [22]. This classification is used today for both traumatic and non-traumatic OCLTs. Furthermore, the ICRS classification is widely used for all cartilage lesions and not only lesions of the ankle joint. In contrast, Giannini et al. presented a specific classification for OCLTs [20]. It takes the status of both bone and cartilage into account and provides a recommendation for therapy for the respective stage. According to this classification, retrograde drilling is recommended only in case of intact overlaying cartilage. The ICRS also mentions intact overlaying cartilage as a precondition for retrograde drilling [18]. In our patients, the cartilage in arthroscopy was often observed to be soft, which corresponds to at least ICRS stage 1. This was not an exclusion criterion for retrograde drilling in our patients. The preoperative ICRS stages in MRI were often lower than the intraoperative ICRS stages in arthroscopy. In addition, Giannini et al. defined the threshold for the decision between microfracture and cartilage replacement therapy at a defect size of 1.5 cm^2^, similar to other guidelines [10]. In contrast, the ICRS consensus suggests that debridement, curettage and BMS is appropriate in OCLA smaller than 1 cm^2^ in size and 5 mm in depth; in OCLA larger than 1 cm^2^, scaffold-based therapies (e.g. ACI or M-BMS) should be used [17, 19]. When applying these thresholds, one should take into account that the defect size is an approximate value. This applies to both MRI and intraoperative findings on arthroscopy.

We used the MOCART 2.0 score, which is an update of the MOCART score; it uses values between 0 and 100 to evaluate the postoperative MRI. This score was developed for the cartilage evaluation of the knee after ACI but can also be used after cartilage repair of the ankle [21]. The mean MOCART 2.0 score in our population was 48.2, whereas the median time between the operation and MRI was 13 months. Remarkably, patients who received a re-operation had a slightly better mean MOCART 2.0 score than patients without re-operation. In a recent series of 22 patients with OCLTs (mean age 14.4 years) who were treated with microfracture and/or drilling and had a mean MRI follow-up period of 8.3 years, Carlson et al. reported a mean postoperative MOCART score of 48 [27]. Jurina et al. also used the MOCART score to evaluate the postoperative status of 11 ankles of skeletally immature patients with OCLTs after microfracture [28]. After a median follow-up of 6.5 years, the overall MOCART score was 65 (range 10–75). Carlson et al. and Jurina et al. did not report an association between MRI results according to the MOCART score and clinical outcome measures. Although the time between surgery and MRI differed between the studies, the MOCART 2.0 scores in the present study are comparable with the MOCART scores reported by the studies mentioned above. In addition, there were no significant differences between patients with and without re-operation with regard to the variables examined in the pre- and postoperative MRI scans. Thus, we could not detect any prognostic factors for a later re-operation based on MRI.

After surgical treatment of OCLTs in children and adolescents, regular clinical follow-ups should be performed. Specific scores can be used pre- and postoperatively (e.g. AOFAS score) to evaluate the individual therapy success. The ICRS recommends performing cross-sectional imaging in patients who remain symptomatic after surgery [29]. Multicentre online registries using patient-reported outcomes measures (PROMs) are suitable for the evaluation of long-term outcomes [30]. Established PROMs such as the Foot and Ankle Outcome Score (FAOS), the Foot and Ankle Ability Measure (FAAM) and the visual analogue scale for pain (VAS) could also be used by older children and adolescents.

Our study has several limitations. According to the surgical techniques used, the study population is heterogeneous. Several surgeons were involved over a long period of time, which is why the treatment decisions might not be uniform. Due to the rarity of OCLT surgery in children, the number of reported cases is small. However, our study includes a higher sample size than the majority of available studies on the topic, with a high follow-up period. Furthermore, the study did not aim to compare different surgical techniques.

The available MRI data were obtained from different scanners with different protocols. Moreover, MRI is known to be inferior to MR arthrography or CT arthrography regarding the classification of chondral lesions. Nevertheless, after conventional radiography, MRI is presumed to be the standard modality in the imaging diagnostic tests used for OCLTs [31]. In our study, only 17 patients could be followed-up with MRI, which represents a selection bias because satisfied patients did not potentially need postoperative MRI and therefore withdraw. This could have negatively influenced the mean value of the MOCART 2.0 score of the total study population, especially in the group of patients without a re-operation.

## Conclusion

We detected a re-operation rate of 25.9% after primary surgical OCLT treatment in paediatric and adolescent patients. Patients with re-operation had significantly lower ICRS classification stages compared to patients without re-operation. Future studies should compare different surgical therapies in terms of indications, re-operation rates and postoperative outcomes. Due to the rarity of OCLTs in children, a multicentre approach should be used, either as a prospective study or a systematic recording in a multicentre registry.

## Data Availability

The datasets generated and/or analysed during the current study are not publicly available due German and European data protection laws.

## Abbreviations

ACI: Autologous chondrocyte implantation
BHL: Berndt-Harty-Loomer
BMI: Body mass index
BMS: Bone marrow stimulation
CT: Computed tomography
FAAM: Foot and Ankle Ability Measure
FAOS: Foot and Ankle Outcome Score
ICRS: International Cartilage Repair Society
M-BMS: Matrix-induced bone marrow stimulation
MOCART: Magnetic Resonance Observation of Cartilage Repair Tissue score
MRI: Magnetic resonance imaging
OCLA: Osteochondral lesion of the ankle
OCLT: Osteochondral lesion of the talus
PROM: Patient-reported outcome Measure
VAS: Visual analogue scale
